# Two-Time-Point Inflammatory Status Patterns and the Risk of Incident Diabetes: A Cohort Study Based on CHARLS

**DOI:** 10.3390/healthcare14152404

**Published:** 2026-08-05

**Authors:** Yanxiang Luo, Han Li, Hongjian Gong, Lixin Guo, Qi Pan

**Affiliations:** 1 Peking University Fifth School of Clinical Medicine, Beijing 100730, China; luoyanxiang1227@163.com; 2 Department of Endocrinology, Beijing Hospital, National Center for Gerontology, National Clinical Research Center for Gerontology, The Key Laboratory of Geriatrics of NHC, Institute of Geriatric Medicine, Chinese Academy of Medical Sciences, Beijing 100730, China; liihan0411@student.pumc.edu.cn (H.L.); b2025021021@student.pumc.edu.cn (H.G.); 3 Graduate School of Peking Union Medical College, Chinese Academy of Medical Sciences, Beijing 100730, China

**Keywords:** diabetes mellitus, inflammatory score, longitudinal study, targeted maximum likelihood estimation

## Abstract

**Background:** Chronic inflammation plays a central role in the pathogenesis of diabetes mellitus (DM). Aims: This study delineated two-time-point inflammatory status patterns and examined their relationship with incident DM risk using data from the China Health and Retirement Longitudinal Study (CHARLS). **Methods:** The temporal changes in the inflammatory score between Wave 1 (2011) and Wave 3 (2015), which was calculated as the sum of Z-scores for C-reactive protein (CRP) and white blood cell (WBC) counts, were specified as the exposure. Incident DM was assessed at Wave 3 (2015) and Wave 4 (2018). Associations between inflammatory status patterns and incident DM was analyzed using a longitudinal targeted maximum likelihood estimation (LTMLE) framework in a cohort study. **Results:** A total of 4305 participants were included, 2404 (55.84%) of whom were female, with a median baseline age of 58 years. During a 7-year follow-up period, 730 individuals (17.0%) developed incident DM. Four distinct inflammatory status patterns were identified: low-stable, high-stable, increasing, and decreasing. After adjusting for multiple covariates, LTMLE analyses showed that the high-stable pattern was significantly associated with an increased risk of DM compared with the low-stable reference group (risk ratio [RR] = 1.430, 95% confidence interval [CI]: 1.204–1.698), with an E-value 2.21. In a landmark analysis of incident DM at Wave 4 (n = 150), the estimates for the high-stable pattern remained directionally positive across the adjusted models but were imprecise and compatible with no association. Exploratory subgroup analyses showed no statistical evidence of effect modification, as neither multiplicative nor additive interaction tests were statistically significant. **Conclusions:** A persistently elevated inflammatory score is significantly associated with a heightened risk of incident DM.

## 1. Introduction

Diabetes mellitus (DM) has emerged as a global health crisis. According to the International Diabetes Federation, the 11th Diabetes Atlas estimated that 589 million adults aged 20–79 years were living with diabetes in 2024, accounting for 11.1% of the global population in this age group [1]. In China, the burden of diabetes is particularly striking, with an estimated 233 million individuals affected in 2023, underscoring the significant public health challenge posed by this chronic disease [2]. Although the pathophysiology of DM is complex, inflammatory pathways have been identified as a unifying underlying mechanism. Chronic inflammation is thought to contribute to the development of DM by inducing β-cell destruction and insulin resistance, while hyperglycemia further exacerbates inflammatory responses, perpetuating a vicious cycle that drives long-term complications [3].

Many key inflammation biomarkers are involved in the occurrence of DM, including white blood cell (WBC) counts, C-reactive protein (CRP), and cytokines, all of which are easily detected in clinical practice. Elevated WBC counts have been consistently associated with an increased risk of DM [4]. Similarly, higher levels of neutrophils and lymphocytes are predictive of DM incidence [5]. In addition to WBC counts, cytokines such as interleukin-6 (IL-6), tumor necrosis factor-alpha, and high-sensitivity CRP (hsCRP) have been identified as potent predictors of DM development [6,7,8]. These markers not only reflect systemic inflammation but also provide insights into the inflammatory burden that contributes to DM. To overcome the limitations of assessing individual markers, researchers have proposed an inflammatory score, which is calculated by combining the Z-scores of WBC and CRP [9,10]. This composite index offers a more systematic and comprehensive assessment of inflammatory status [9,10], providing a reliable metric for evaluating inflammatory burden.

Despite advances in understanding the role of inflammation in DM, most studies have relied on single-time point assessments of inflammatory markers [11]. Such approaches fail to capture the temporal dynamics of inflammation, which can fluctuate over time—either increasing, remaining stable, or decreasing. This study addresses these gaps by employing a longitudinal approach, utilizing the Longitudinal Targeted Maximum Likelihood Estimation (LTMLE) framework [12,13,14]. By evaluating the change in the inflammatory score over time, we aim to estimate the association of chronic inflammation and the development and progression of DM.

## 2. Methods

### 2.1. Data Source and Study Population

**Figure 1:**
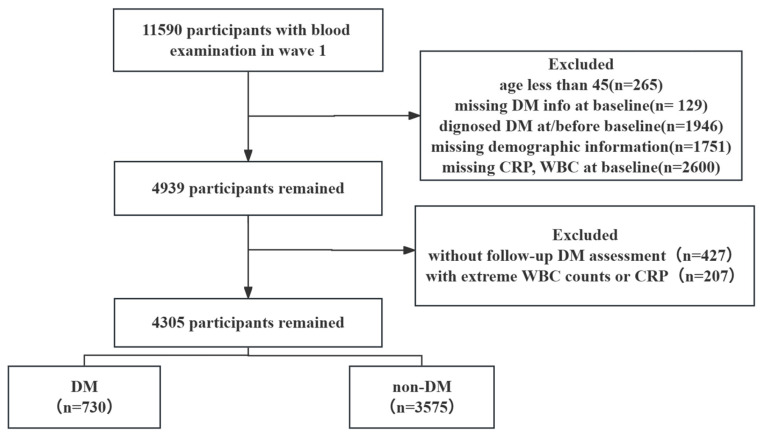
**Flowchart for the selection of participants in the cohort study (CHARLS, 2011–2018).** Abbreviations: DM, diabetes mellitus; CRP, C-reactive protein; WBC, white blood cell.

The study utilized data from the China Health and Retirement Longitudinal Study (CHARLS), a nationally representative cohort of Chinese adults. Participants were enrolled at the baseline survey in 2011 from 150 counties or districts using multistage probability sampling. Surveys were conducted every 2 years in 2013, 2015, 2018, and 2020, representing Wave 2, Wave 3, Wave 4, and Wave 5, respectively. Blood collection was performed at Wave 1 and Wave 3. From an initial pool of 11,590 participants with baseline biomarker data, we sequentially excluded participants aged < 45 years (n = 265); missing DM status at baseline (n = 129) or missing demographic variables and comorbidities at baseline (n = 1751); without complete WBC counts or CRP data at baseline (n = 2600); diagnosed with DM at baseline (n = 1946); with extreme WBC counts or CRP (exceeding three standard deviations from the mean; n = 207); and without follow-up DM assessment (n = 427), resulting in an analytical cohort of 4305 individuals (Figure 1). The study received approval from Peking University’s Institutional Review Board (IRB00001052-11015) [15], obtained written informed consent from all participants, and adhered to STROBE reporting guidelines [16].

### 2.2. Exposure Definition

Longitudinal inflammatory status was assessed using a composite score derived from Wave 1 (2011) and Wave 3 (2015) data. CRP values were used on their original scale and were not logarithmically transformed before standardization. At each wave, the Z-score for each biomarker was calculated using the following formula:

$$Z=\frac{X-M}{SD}$$

where *X* represents the individual biomarker value, *M* represents the wave-specific mean, and *SD* represents the wave-specific standard deviation. CRP and WBC showed statistically significant positive correlations at both measurement waves (Wave 1: Spearman’s ρ = 0.201, *p* < 0.001; Wave 3: ρ = 0.242, *p* < 0.001). To integrate information from these complementary inflammatory markers, the inflammatory score was subsequently calculated as the sum of the CRP and WBC Z-scores:

Inflammatory score = Z_CRP_ + Z_WBC_

### 2.3. Ascertainment of Incident DM

DM was diagnosed when the patient met any of the following criteria: physician-diagnosed DM by self-report, fasting glucose (FBG) ≥ 7.0 mmol/L, or hemoglobin A1c (HbA1c) ≥ 6.5%. Participants with prevalent DM at baseline were excluded from follow-up ascertainment. Incident DM was coded as a binary outcome (1 = incident case; 0 = non-case), which was determined at Wave 3 (2015) and Wave 4 (2018).

### 2.4. Covariates

We incorporated covariates measured at Wave 1, including demographic factors, anthropometric data, comorbidities and serum biomarkers.

### 2.5. Statistical Analysis

Descriptive statistics were used to characterize participants, with normally distributed continuous variables presented as means ± standard deviations, skewed continuous variables as medians (interquartile ranges [IQR]), and categorical variables as frequencies (percentages). Inflammatory status patterns were modeled across Waves 1 and 3 using the longitudinal inflammatory score. Given the absence of clinical cutoffs, we defined “elevated inflammation” as values exceeding the cohort median at each wave. Participants’ inflammation changes were categorized into four mutually exclusive patterns (Supplementary Table S1 and Supplementary Figure S1): low-stable, high-stable, increasing, and decreasing. Extreme values of continuous variables, including age, BMI, blood pressure, and serum biomarkers, were winsorized. Analyses accounted for missing data through multiple imputation using the R mice package (Supplementary Table S2).

The association between inflammatory status patterns and incident DM was evaluated using LTMLE as implemented in the ltmle package. The observed longitudinal data structure was represented as (O = (W, A1, A2, Y)), where W denoted baseline covariates measured at Wave 1, A1 and A2 denoted inflammatory status measured at Waves 1 and 3 and were specified as the exposure nodes, and Y denoted incident DM assessed at Waves 3 and 4 and was specified as the outcome node. No time-varying covariate nodes or censoring models were included. The analysis was restricted to participants with the follow-up information. LTMLE was performed separately within each imputed dataset, and the resulting effect estimates and corresponding within-imputation variances were combined using Rubin’s rules.

Five covariate adjustment models were hierarchically constructed: Model 1 (age, sex); Model 2 (Model 1 + education, hukou, marital status, smoking, alcohol); Model 3 (Model 2 + comorbidities); Model 4 (Model 3 + glucose and glycated hemoglobin); Model 5 (Model 3 + BMI). Additionally, we calculated the E-value to quantify the strength of potential unmeasured confounding required to explain away the observed associations. In addition, results from the LTMLE were compared with those obtained using the G-computation method, and multiple superlearner libraries, including generalized linear models (GLMs), random forests, and XGBoost, were employed to assess the stability of the results (see Supplementary Materials for details).

Subgroup analyses, stratified by sex, BMI (<25/≥25 kg/m^2^), residence (agriculture/non-agriculture), smoking status (yes/no), and alcohol status (yes/no), were conducted to assess the effect heterogeneity. Additive and multiplicative interactions were formally tested. Statistical significance was established at two-sided *p* < 0.05, with all analyses conducted in R version 4.5.0.

## 3. Results

### 3.1. Clinical Characteristics of the Study Population

The study included 4305 participants with a median age of 58 years (IQR: 52–64), 2404 (55.84%) of whom were female at baseline. During follow-up, 730 participants developed incident DM. The baseline characteristics are detailed in Table 1. The median (IQR) inflammatory score was −0.27 (−1.02 to 0.68) at baseline and −0.26 (−1.07 to 0.74) at the second assessment. The participants were dichotomized at the cohort median, with individuals who developed DM showing higher inflammatory scores at both time points than those without DM (time point 1: median [IQR] −0.02 [−0.86, 0.90] vs. −0.31 [−1.04, 0.63]; time point 2: 0.05 [−0.75, 1.21] vs. −0.32 [−1.12, 0.65]; both *p* < 0.001). Additionally, participants with DM were older and had a higher prevalence of hypertension, dyslipidemia, and cardiovascular disease, as well as higher blood pressure, BMI, waist circumference, blood glucose, total cholesterol, triglycerides, low-density lipoprotein, and uric acid levels compared with those without DM at baseline (all *p* < 0.001).

### 3.2. Different Inflammatory Status Patterns

Four distinct inflammatory status patterns were identified for inflammatory score: low-stable, decreasing, increasing, and high-stable (Supplementary Table S1). The baseline characteristics by inflammatory status patterns are presented in Table 2. The proportion of female participants decreased progressively from the low-stable (62.96%) to the high-stable group (51.17%). BMI, waist circumference and blood pressure demonstrated positive trends across different groups (all *p* < 0.001). Higher proportions of current smokers and alcohol consumers more than once a month were observed in the high-stable, increasing, and decreasing groups relative to the low-stable group.

### 3.3. Association Between Inflammatory Status Patterns and DM Risk

The LTMLE results are shown in Table 3. After progressive adjustment for covariates, the high-stable group was consistently associated with a significantly higher risk of incident DM than the low-stable reference group. In the model 5, the RR was 1.430 (95% CI: 1.204–1.698; *p* < 0.001). In contrast, neither the increasing nor the decreasing group was significantly associated with incident DM in the LTMLE analyses. In Model 5, the RRs were 1.076 (95% CI: 0.870–1.330; *p* = 0.499) for the increasing group and 1.000 (95% CI: 0.803–1.246; *p* = 0.998) for the decreasing group.

### 3.4. Sensitivity Analyses

An additional sensitivity analysis was performed using the G-computation method, and its results were largely consistent with those obtained from the LTMLE method (Table 3). Across Models 1–5, the high-stable group was consistently associated with a significantly higher risk of incident DM than the low-stable reference group. G-computation also indicated an elevated risk in the increasing group across all models. In Model 5, the RRs were 1.473 (95% CI: 1.225–1.771; *p* < 0.001) for the high-stable group and 1.266 (95% CI: 1.026–1.562; *p* = 0.028) for the increasing group. Furthermore, E-value was calculated to assess the potential influence of unmeasured confounding. In the LTMLE analysis, the E-value for the point estimate of the high-stable group in Model 5 exceeded 2.0 (E-value = 2.21), while the E-value for the 95% confidence limit closest to the null was 1.70. The corresponding point-estimate E-values for the decreasing and increasing patterns were 1.02 and 1.36, respectively. Similarly, the corresponding point-estimate E-values from the G-computation analysis were 1.62, 1.85, and 2.31 for the decreasing, increasing, and high-stable groups, respectively.

Furthermore, the results derived from different superlearner libraries were generally consistent with those of the LTMLE method (Supplementary Tables S3 and S4). These findings suggest that the high-stable group is associated with an increased risk of DM and that this association is relatively robust to unmeasured confounding. In contrast, the associations for the increasing status pattern of inflammation may be more susceptible to the choice of estimation method. Moreover, in a landmark analysis excluding diabetes cases identified at Wave 3 (150 incident DM events at Wave 4), the estimates for the high-stable pattern remained above 1 across the adjusted models, but the confidence intervals were wide and included the null (Supplementary Table S5).

### 3.5. Subgroup Analyses and Interactions

**Figure 2:**
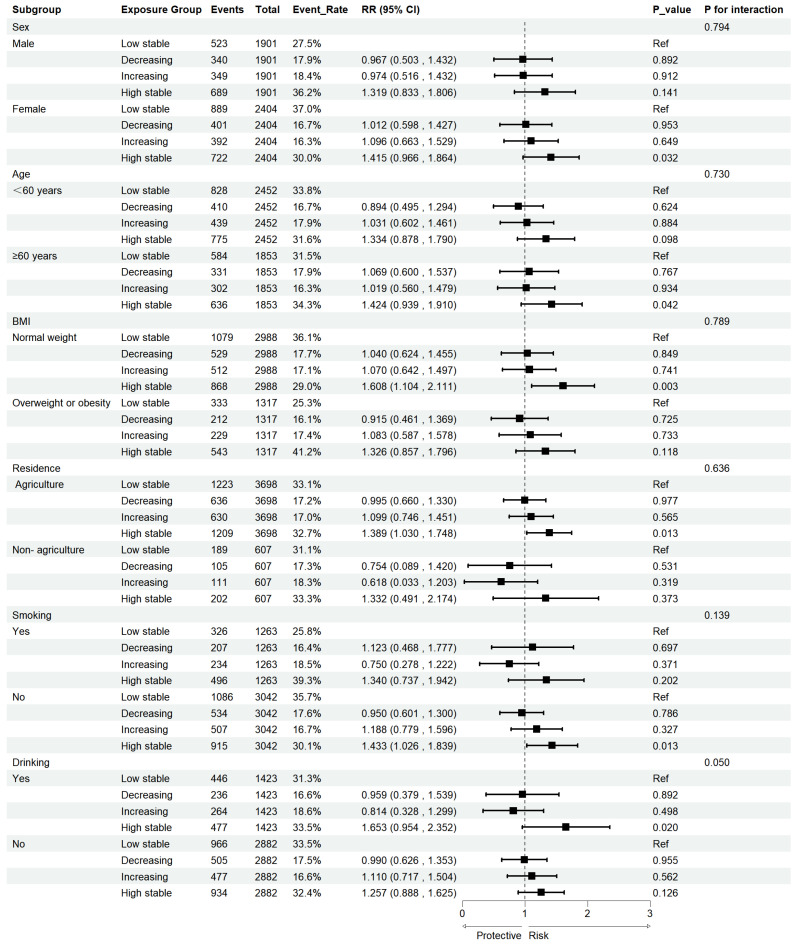
**Subgroup analyses of the association between inflammatory status patterns and incident DM.** RR and 95% CI were obtained from LTMLE, and all models were adjusted for age, gender, residence, education, marital, smoking, drinking, hypertension, dyslipidemia, cardiovascular disease, liver disease, chronic kidney disease and BMI. All covariates were assessed at baseline. RR, Risk ratio; CI, confidence interval; BMI, Body mass index; Ref, reference.

Forest plots of the exploratory subgroup analyses are presented in Figure 2. The high-stable group was associated with significantly elevated risks among individuals with BMI < 25 kg/m^2^ (RR = 1.608, 95% CI: 1.104–2.111), rural residents (RR = 1.389, 95% CI: 1.030–1.748), and non-smokers (RR = 1.433, 95% CI: 1.026–1.839). No significant risks were observed in the overweight/obese, non-rural, or smoking subgroups. Neither decreasing nor increasing groups showed significant risk associations across any subgroups. Multiplicative and additive interaction tests indicated no statistically significant interactions between inflammatory status patterns and stratification factors (all interaction *p* > 0.05).

## 4. Discussion

Inflammation is associated with the development of DM. In this study, LTMLE analysis showed that, in a study population characterized by relatively good health and high adherence to follow-up, individuals with a stably high inflammatory score exhibited a significantly increased risk of incident DM (RR 1.430, 95% CI: 1.204–1.698) compared with those with a stably low score. Moreover, the E-values for the point estimate and the lower confidence limit were 2.21 and 1.70, respectively, indicating the strength of uncontrolled confounding required to move the estimate toward the null. In a landmark analysis excluding participants with diabetes identified at Wave 3, leaving 150 incident DM events at Wave 4, the estimates for the high-stable pattern remained directionally positive across the adjusted models but were imprecise and compatible with no association. Exploratory subgroup analyses showed no statistical evidence of effect modification, as neither multiplicative nor additive interaction tests were statistically significant.

Previous studies have demonstrated that the cumulative burden of elevated inflammation indices significantly enhances the risk of DM. For instance, the time-averaged cumulative CRP levels were significantly associated with incident DM in a real-world, prospective cohort [17]. Additionally, a standard deviation increase in the cumulative remnant cholesterol inflammation index was associated with a 1.26-fold higher OR for developing diabetes [18]. Although these studies did not focus on the temporal variations in inflammation, these findings collectively reinforce our conclusion that a sustained elevation of inflammatory score may be associated with a higher risk of incident DM. Moreover, this association remained statistically significant in separate models additionally adjusted for glucose and glycated hemoglobin (Model 4) and for BMI (Model 5). Future studies should investigate whether longitudinal inflammatory patterns provide incremental predictive value beyond these conventional risk factors.

Delving into the underlying mechanisms, inflammation is a pivotal driver in the pathogenesis and progression of DM. In type 2 diabetes mellitus (T2DM), nutrient overload, a hallmark of obesity, instigates this process by inducing oxidative and endoplasmic reticulum stress. Metabolites such as glucose and free fatty acids then activate key inflammatory pathways, leading to the production of pro-inflammatory cytokines. They impair insulin signaling and promote pro-inflammatory polarization of adipose tissue macrophages, forming a self-sustaining inflammatory circuit that accelerates β-cell dysfunction [19,20]. Concurrently, unhealthy diets disrupt gut microbiota composition, leading to dysbiosis. The microbial products activate local immune responses, further eroding tight junctions and facilitating the translocation of bacteria and metabolites into circulation, thereby propagating systemic inflammation [21]. Furthermore, environmental factors can epigenetically reprogram immune cells toward a pro-inflammatory phenotype, thereby priming a state of chronic, low-grade inflammation [22]. In type 1 diabetes mellitus (T1DM), a primary insult to the pancreatic islets may activate innate immunity and initiate an inflammatory cascade, ultimately contributing to autoimmune β-cell destruction [23]. Collectively, these mechanisms explain the observed link between the inflammatory status patterns and DM risk. The stably high inflammatory status pattern promotes the accumulation of pro-inflammatory mediators, thereby disrupting insulin signaling and accelerating pathogenesis.

Overweight/obesity (high BMI) is an independent and dose-dependent risk factor for DM [24]. Specifically, obesity drives chronic inflammation via pro-inflammatory cytokines, leading to insulin resistance and β-cell dysfunction. [20,25,26]. Similarly, smoking has been identified as an independent risk factor for DM [27,28], with CRP potentially mediating this association [29]; this occurs as tobacco use amplifies inflammation and oxidative stress, leading to tissue injury and metabolic dysfunction [30]. In the rural subgroup, a distinct set of environmental and psychosocial exposures reinforced the link between inflammation and DM [31]. In rural China, household air pollution represents a major disease burden [32] and has been linked to systemic inflammatory responses [33]. Furthermore, risk factors for DM, such as hypertension and mental health disorders, were found to be more pronounced in rural regions [34]. Moreover, poor psychological environments and chronic stressors further contribute to elevated inflammation [35]. Collectively, these observations suggest that obesity, smoking, and factors associated with rural residence may be associated with both systemic inflammation and DM risk. However, because no statistically significant effect modification was detected, these explanations remain speculative and require evaluation in future adequately powered studies.

Our study has several notable strengths. First, by capturing the dynamic changes in inflammatory score over time, it addresses a key limitation inherent to studies that rely solely on single-time-point, cross-sectional measurements. Second, the application of LTMLE enabled doubly robust estimation of standardized marginal risks across longitudinal inflammatory status patterns.

However, several limitations should be considered. First, the study population consisted exclusively of Chinese adults, which may limit the generalizability of our findings to other racial and ethnic populations. Second, several methodological choices—including the lack of log transformation of CRP, the use of conventional rather than high-sensitivity CRP, median-based dichotomization, and wave-specific standardization—may have influenced the estimates. In particular, wave-specific standardization may reflect relative rather than absolute biological changes, while excluding extreme CRP and WBC values may have removed both acute inflammatory observations and individuals with genuinely high chronic inflammation. Third, inflammation was assessed at only two time points, additional inflammatory biomarkers were unavailable [36,37], and residual confounding from family history, medication use, and diet remains possible. Fourth, the second inflammatory measurement and diabetes ascertainment occurred in the same wave for most cases, leaving the possibility of reverse causation. The landmark analysis included only 150 events and yielded an estimate compatible with the null. Finally, more than half of the initially eligible biomarker sample was excluded, and loss to follow-up was not modeled through a censoring mechanism, potentially introducing selection bias.

## 5. Conclusions

In summary, this study identified an association between longitudinal patterns of inflammatory status and incident DM. These findings may provide further insight into the role of chronic inflammation in the development of DM.

## Figures and Tables

**Table 1:** Comparison of participant characteristics by incident diabetes status.

Attributes	Characteristics	Time Points	Incident Diabetes (n = 730)	No Incident Diabetes (n = 3575)	*p* Value
Binary exposure	Inflammatory scores = High (%)	Time point 1	423 (57.95%)	1729 (48.36%)	<0.001
		Time point 2	433 (59.32%)	1719 (48.08%)	<0.001
	Inflammatory scores (median (IQR))	Time point 1	−0.02 (−0.86, 0.90)	−0.31 (−1.04, 0.63)	<0.001
		Time point 2	0.05 (−0.75, 1.21)	−0.32 (−1.12, 0.65)	<0.001
Covariates at baseline	Gender = Female (%)		419 (57.40%)	1985 (55.52%)	0.375
	Education (%)				0.365
	Primary school		672 (92.05%)	3231 (90.38%)	
	Secondary school		51 (6.99%)	302 (8.45%)	
	Tertiary school		7 (0.96%)	42 (1.17%)	
	Residence (%)				0.940
	Agricultural		627 (85.89%)	3071 (85.90%)	
	Non agricultural		100 (13.70%)	484 (13.54%)	
	Unified residence		3 (0.41%)	20 (0.56%)	
	Marital status (%)				0.077
	Married		645 (88.36%)	3238 (90.57%)	
	Not married or widowed		85 (11.64%)	337 (9.43%)	
	Smoking status (%)				0.700
	Current smokers		205 (28.08%)	1058 (29.59%)	
	Former smokers		58 (7.95%)	286 (8.00%)	
	Never smokers		467 (63.97%)	2231 (62.41%)	
	Drinking status (%)				0.361
	Drink more than once a month		169 (23.15%)	909 (25.43%)	
	Drink less than once a month		56 (7.67%)	289 (8.08%)	
	None of these		505 (69.18%)	2377 (66.49%)	
	Hypertension, n (%)		339 (46.44%)	1237 (34.60%)	<0.001
	Dyslipidemia, n (%)		91 (12.47%)	274 (7.66%)	<0.001
	cardiovascular disease, n (%)		122 (16.71%)	407 (11.38%)	<0.001
	Liver disease, n (%)		35 (4.79%)	131 (3.66%)	0.180
	Chronic kidney disease, n (%)		55 (7.53%)	218 (6.10%)	0.171
	Age (median (IQR))		59.00 (53.00, 65.00)	58.00 (51.00, 64.00)	<0.001
	BMI kg/m^2^ (median (IQR))		24.20 (21.72, 26.90)	22.97 (20.80, 25.40)	<0.001
	Waist cm (median (IQR))		88.00 (80.00, 94.60)	83.80 (77.20, 90.20)	<0.001
	SBP mmHg (median (IQR))		128.00 (116.50, 142.50)	123.50 (111.50, 138.00)	<0.001
	DBP mmHg (median (IQR))		75.50 (68.00, 84.00)	73.50 (66.00, 82.00)	<0.001
	Glucose mg/dL (median (IQR))		104.76 (96.97, 112.32)	99.54 (92.88, 106.38)	<0.001
	Glycated Hemoglobin % (median (IQR))		5.20 (5.00, 5.50)	5.10 (4.80, 5.30)	<0.001
	Cholesterol mg/dL (median (IQR))		195.04 (172.04, 219.59)	188.27 (166.24, 212.24)	<0.001
	TG mg/dL (median (IQR))		115.49 (81.20, 170.14)	99.12 (71.68, 141.60)	<0.001
	HDL mg/dL (median (IQR))		47.55 (38.66, 57.99)	51.03 (42.14, 61.08)	<0.001
	LDL mg/dL (median (IQR))		117.53 (97.04, 141.11)	113.66 (93.17, 135.31)	0.003
	Uric Acid mg/dL (median (IQR))		4.33 (3.58, 5.18)	4.15 (3.49, 4.97)	0.001
	Creatinine mg/dL (median (IQR))		0.76 (0.64, 0.87)	0.75 (0.64, 0.86)	0.538

Note: (1) Continuous variables were expressed as mean ± SD or median (IQR). Categorical variables were expressed as frequencies (percentages). (2) Abbreviation: BMI, Body mass index; SBP, Systolic blood pressure; DBP, Diastolic blood pressure; TG, Triglycerides; HDL, High-density lipoprotein; LDL, Low-density lipoprotein; DM: Diabetes mellitus.

**Table 2:** Characteristics of the study population with different inflammatory status patterns.

Characteristics	Low Stable (n = 1412)	Decreasing (n = 741)	Increasing (n = 741)	High Stable (n = 1411)	*p* Value
Gender = Female (%)	889 (62.96%)	401 (54.12%)	392 (52.90%)	722 (51.17%)	<0.001
Education (%)					0.291
Primary school	1279 (90.58%)	663 (89.47%)	665 (89.74%)	1296 (91.85%)	
Secondary school	112 (7.93%)	68 (9.18%)	69 (9.31%)	104 (7.37%)	
Tertiary school	21 (1.49%)	10 (1.35%)	7 (0.94%)	11 (0.78%)	
Residence (%)					0.939
Agricultural	1223 (86.61%)	636 (85.83%)	630 (85.02%)	1209 (85.68%)	
Non agricultural	183 (12.96%)	100 (13.50%)	106 (14.30%)	195 (13.82%)	
Unified residence	6 (0.42%)	5 (0.67%)	5 (0.67%)	7 (0.50%)	
Marital status (%)					0.610
Married	1278 (90.51%)	665 (89.74%)	676 (91.23%)	1264 (89.58%)	
Not married or widowed	134 (9.49%)	76 (10.26%)	65 (8.77%)	147 (10.42%)	
Smoking status (%)					<0.001
Current smokers	326 (23.09%)	207 (27.94%)	234 (31.58%)	496 (35.15%)	
Former smokers	92 (6.52%)	64 (8.64%)	63 (8.50%)	125 (8.86%)	
Never smokers	994 (70.40%)	470 (63.43%)	444 (59.92%)	790 (55.99%)	
Drinking status (%)					0.040
Drinking more than once a month	321 (22.73%)	172 (23.21%)	210 (28.34%)	375 (26.58%)	
Drinking less than once a month	125 (8.85%)	64 (8.64%)	54 (7.29%)	102 (7.23%)	
None of these	966 (68.41%)	505 (68.15%)	477 (64.37%)	934 (66.19%)	
Hypertension, n (%)	403 (28.54%)	258 (34.82%)	281 (37.92%)	634 (44.93%)	<0.001
Dyslipidemia, n (%)	104 (7.37%)	56 (7.56%)	66 (8.91%)	139 (9.85%)	0.083
cardiovascular disease, n (%)	168 (11.90%)	81 (10.93%)	95 (12.82%)	185 (13.11%)	0.467
Liver disease, n (%)	70 (4.96%)	28 (3.78%)	25 (3.37%)	43 (3.05%)	0.055
Chronic kidney disease, n (%)	92 (6.52%)	50 (6.75%)	47 (6.34%)	84 (5.95%)	0.888
Age (median (IQR))	57.00 (51.00, 64.00)	58.00 (52.00, 65.00)	58.00 (52.00, 63.00)	59.00 (52.00, 64.00)	0.100
BMI kg/m^2^ (median (IQR))	22.48 (20.58, 24.77)	22.97 (20.55, 25.31)	23.14 (20.99, 25.78)	23.88 (21.57, 26.54)	<0.001
Waist cm (median (IQR))	82.00 (76.00, 89.00)	83.20 (76.80, 89.80)	84.20 (78.00, 91.80)	87.00 (80.00, 94.00)	<0.001
SBP mmHg (median (IQR))	121.00 (110.00, 135.00)	124.00 (111.50, 137.00)	124.00 (112.00, 140.00)	127.50 (115.50, 142.50)	<0.001
DBP mmHg (median (IQR))	71.50 (65.00, 80.00)	74.00 (66.00, 81.50)	74.00 (67.00, 82.50)	75.50 (68.00, 84.50)	<0.001
Glucose mg/dL (median (IQR))	99.18 (92.52, 105.84)	100.62 (94.09, 106.96)	99.18 (92.16, 106.74)	101.88 (94.50, 109.62)	<0.001
Glycated Hemoglobin % (median (IQR))	5.00 (4.80, 5.30)	5.10 (4.90, 5.40)	5.10 (4.80, 5.30)	5.10 (4.90, 5.40)	<0.001
Cholesterol mg/dL (median (IQR))	185.95 (163.53, 207.60)	187.50 (165.08, 212.05)	188.66 (167.40, 213.79)	194.85 (171.65, 219.20)	<0.001
TG mg/dL (median (IQR))	92.04 (68.14, 128.32)	97.35 (69.03, 138.50)	101.78 (71.68, 144.26)	115.93 (81.42, 169.92)	<0.001
HDL mg/dL (median (IQR))	52.96 (44.46, 63.02)	51.42 (42.91, 61.47)	49.87 (40.98, 60.31)	46.78 (38.27, 56.44)	<0.001
LDL mg/dL (median (IQR))	111.73 (91.53, 132.99)	110.18 (90.85, 134.25)	113.27 (94.72, 134.92)	118.11 (97.13, 140.63)	<0.001
Uric Acid mg/dL (median (IQR))	3.92 (3.27, 4.67)	4.21 (3.52, 5.08)	4.13 (3.49, 4.97)	4.47 (3.77, 5.30)	<0.001
Creatinine mg/dL (median (IQR))	0.72 (0.63, 0.84)	0.75 (0.64, 0.87)	0.75 (0.66, 0.86)	0.76 (0.67, 0.89)	<0.001

Note: (1) Continuous variables were expressed as mean ± SD or median (IQR). Categorical variables were expressed as frequencies (percentages). (2) Abbreviation: BMI, Body mass index; SBP, Systolic blood pressure; DBP, Diastolic blood pressure; TG, Triglycerides; HDL, High-density lipoprotein; LDL, Low-density lipoprotein; DM: Diabetes mellitus.

**Table 3:** Comparison of the effects of inflammatory status patterns on incident DM risk using ltmle and g-computation methods with generalized linear model.

			Standardised Marginal Risk for the Inflammatory Pattern (%)	Standardised Marginal Risk for the Low-Stable Reference Pattern (%)	RR	CI Low	CI High	*p* Value
Model 1	Decreasing	LTMLE	14.54	13.45	1.081	0.868	1.346	0.488
		G-computation	15.84	12.61	1.256	1.014	1.556	0.037
	Increasing	LTMLE	16.04	13.45	1.192	0.964	1.473	0.105
		G-computation	17.49	12.61	1.387	1.127	1.707	0.002
	High stable	LTMLE	22.42	13.45	1.666	1.412	1.966	<0.001
		G-computation	21.65	12.61	1.717	1.442	2.045	<0.001
Model 2	Decreasing	LTMLE	14.45	13.52	1.069	0.858	1.333	0.552
		G-computation	15.80	12.59	1.255	1.012	1.557	0.039
	Increasing	LTMLE	16.11	13.52	1.192	0.964	1.474	0.105
		G-computation	17.53	12.59	1.393	1.130	1.717	0.002
	High stable	LTMLE	22.64	13.52	1.675	1.418	1.978	<0.001
		G-computation	21.68	12.59	1.723	1.443	2.056	<0.001
Model 3	Decreasing	LTMLE	14.62	13.97	1.047	0.839	1.306	0.685
		G-computation	15.97	12.94	1.235	0.994	1.534	0.057
	Increasing	LTMLE	15.79	13.97	1.130	0.913	1.400	0.260
		G-computation	17.36	12.94	1.342	1.087	1.655	0.006
	High stable	LTMLE	22.29	13.97	1.596	1.347	1.892	<0.001
		G-computation	21.17	12.94	1.636	1.364	1.963	<0.001
Model 4	Decreasing	LTMLE	14.48	14.88	0.973	0.782	1.211	0.808
		G-computation	15.50	14.01	1.107	0.892	1.373	0.357
	Increasing	LTMLE	16.49	14.88	1.108	0.900	1.363	0.333
		G-computation	17.98	14.01	1.283	1.047	1.573	0.016
	High stable	LTMLE	20.71	14.88	1.392	1.175	1.648	<0.001
		G-computation	19.78	14.01	1.412	1.181	1.688	<0.001
Model 5	Decreasing	LTMLE	14.81	14.81	1.000	0.803	1.246	0.998
		G-computation	16.05	13.69	1.173	0.943	1.457	0.151
	Increasing	LTMLE	15.93	14.81	1.076	0.870	1.330	0.499
		G-computation	17.33	13.69	1.266	1.026	1.562	0.028
	High stable	LTMLE	21.18	14.81	1.430	1.204	1.698	<0.001
		G-computation	20.16	13.69	1.473	1.225	1.771	<0.001

Model 1 adjust: age, gender; Model 2 adjust: age, gender, residence, education, marital, smoking, drinking; Model 3 adjust: age, gender, residence, education, marital, smoking, drinking, hypertension, dyslipidemia, cardiovascular disease, liver disease, chronic kidney disease; Model 4 adjust: age, gender, residence, education, marital, smoking, drinking, hypertension, dyslipidemia, cardiovascular disease, liver disease, chronic kidney disease, glucose, glycated hemoglobin; Model 5 adjust: age, gender, residence, education, marital, smoking, drinking, hypertension, dyslipidemia, cardiovascular disease, liver disease, chronic kidney disease, BMI; All covariates were assessed at baseline. Abbreviation: RR, Risk ratio; CI, Confidence interval; BMI, Body mass index.

## Data Availability

The data used in this study are publicly available from the China Health and Retirement Longi-tudinal Study (CHARLS) website upon registration and approval. Additional study-related mate-rials are available from the corresponding author upon reasonable request.

## Supplementary Materials

The following supporting information can be downloaded at: https://www.mdpi.com/article/10.3390/healthcare14152404/s1; Table S1. Definition of inflammatory status patterns in our study; Table S2. Variables with missing data and the corresponding numbers of missing observations; Table S3. Comparison of the effects of inflammatory status patterns on incident DM risk using ltmle and g-computation methods with superlearner library 1; Table S4. Comparison of the effects of inflammatory status patterns on incident DM risk using ltmle and g-computation methods with superlearner library 2; Table S5. Landmark analysis of the associations between inflammatory status patterns and incident DM using ltmle; Figure S1. Boxplots of inflammatory score, white blood cell count, and C-reactive protein levels across survey waves according to two-time-point inflammatory status patterns; Supplementary File S1: LTMLE model.
