# Supplementary material for: Two-Time-Point Inflammatory Status Patterns and the Risk of Incident Diabetes: A Cohort Study Based on CHARLS
Source: Healthcare (Basel). 2026 Aug 5;14(15):2404. doi: 10.3390/healthcare14152404 (PMC13465524; doi:10.3390/healthcare14152404)
Supplement: Supplementary file 1 [file healthcare-14-02404-s001.zip › healthcare-4430822-supplementary.pdf]

Supplementary Tables

Table S1. Definition of inflammatory status patterns in our study.

Exposure	The first time point	The second time point	Trajectories
Inflammatory score	0	0	Low stable
	1	0	Decreasing
	0	1	Increasing
	1	1	High stable

Table S2. Variables with missing data and the corresponding numbers of missing observations.

Variable	Missing count	Missing rate
BMI kg/m^2（Time point 2）	67	1.5563%
SBP mmHg（Time point 2）	59	1.3705%
DBP mmHg（Time point 2）	59	1.3705%
waist cm（Time point 2）	48	1.1150%
Glycated Hemoglobin %（Time point 1）	19	0.4413%
Hypertension（Time point 2）	16	0.3717%
Glucose mg/dL（Time point 1）	9	0.2091%
Creatinine mg/dl（Time point 1）	9	0.2091%
Creatinine mg/dl（Time point 1）	6	0.1394%
TG mg/dl（Time point 1）	5	0.1161%
smoking status （Time point 2）	3	0.0697%
Glycated Hemoglobin %（Time point 2）	3	0.0697%
drinking status （Time point 2）	2	0.0465%
LDL mg/dl（Time point 1）	2	0.0465%
Glucose mg/dL（Time point 2）	1	0.0232%

Uric Acid mg/dl（Time point 2）	1	0.0232%

Table S3. **Comparison of the effects of inflammatory status patterns on incident DM risk using ltmle and g-computation methods with superlearner library 1.**

			Standardised Marginal Risk For The Inflammatory Pattern(%)	Standardised Marginal Risk For The Low-stable Reference pattern(%)	RR	CI low	CI high	P value
Model 1	Decreasing	LTMLE	14.49	13.48	1.075	0.864	1.337	0.515
		G-computation	15.79	12.64	1.249	1.010	1.546	0.041
	Increasing	LTMLE	16.05	13.48	1.191	0.965	1.470	0.104
		G-computation	17.45	12.64	1.380	1.122	1.697	0.002
	High stable	LTMLE	22.43	13.48	1.664	1.411	1.962	<0.001
		G-computation	21.68	12.64	1.715	1.441	2.041	<0.001
Model 2	Decreasing	LTMLE	14.47	13.53	1.069	0.860	1.329	0.549
		G-computation	15.68	12.68	1.236	0.999	1.531	0.052
	Increasing	LTMLE	16.20	13.53	1.197	0.970	1.476	0.093
		G-computation	17.43	12.68	1.375	1.117	1.691	0.003
	High stable	LTMLE	22.65	13.53	1.673	1.419	1.973	<0.001
		G-computation	21.78	12.68	1.717	1.442	2.045	<0.001
Model 3	Decreasing	LTMLE	14.60	13.95	1.047	0.842	1.301	0.681
		G-computation	15.88	12.99	1.222	0.987	1.513	0.066
	Increasing	LTMLE	15.87	13.95	1.138	0.922	1.403	0.228
		G-computation	17.28	12.99	1.330	1.082	1.636	0.007

	High stable	LTMLE	22.32	13.95	1.599	1.353	1.891	<0.001
		G-computation	21.25	12.99	1.636	1.368	1.955	<0.001
Model 4	Decreasing	LTMLE	14.45	14.89	0.971	0.785	1.200	0.783
		G-computation	15.41	14.09	1.093	0.887	1.348	0.405
	Increasing	LTMLE	16.54	14.89	1.111	0.909	1.357	0.306
		G-computation	17.92	14.09	1.272	1.044	1.549	0.017
	High stable	LTMLE	20.67	14.89	1.388	1.178	1.635	<0.001
		G-computation	19.80	14.09	1.405	1.182	1.670	<0.001
Model 5	Decreasing	LTMLE	14.79	14.77	1.002	0.812	1.237	0.987
		G-computation	16.03	13.69	1.171	0.951	1.442	0.137
	Increasing	LTMLE	15.96	14.77	1.081	0.882	1.324	0.455
		G-computation	17.30	13.69	1.263	1.033	1.546	0.023
	High stable	LTMLE	21.23	14.77	1.438	1.218	1.697	<0.001
		G-computation	20.20	13.69	1.475	1.236	1.761	<0.001

Model 1 adjust: age, gender; Model 2 adjust: age, gender, residence, education, marital, smoking, drinking; Model 3 adjust: age, gender, residence, education, marital, smoking, drinking, hypertension, dyslipidemia, cardiovascular disease, liver disease, chronic kidney disease; Model 4 adjust: age, gender, residence, education, marital, smoking, drinking, hypertension, dyslipidemia, cardiovascular disease, liver disease, chronic kidney disease, glucose, glycated hemoglobin;Model 5 adjust: age, gender, residence, education, marital, smoking, drinking, hypertension, dyslipidemia, cardiovascular disease, liver disease, chronic kidney disease, BMI;All covariates were assessed at baseline.

superlearner library 1 includes generalized linear Model and xgboost.Abbreviation: RR, Risk ratio; CI, Confidence interval; BMI, Body mass index.

Table S4. Comparison of the effects of inflammatory status patterns on incident DM risk using ltmle and g-computation methods with superlearner library 2.

			Standardised Marginal Risk For The	Standardised Marginal Risk For The Low-stable	RR	CI low	CI high	P value

			Inflammatory Pattern(%)	Reference pattern(%)				
Model 1	Decreasing	LTMLE	14.49	13.47	1.075	0.865	1.337	0.513
		G-computation	15.46	12.67	1.220	0.981	1.518	0.074
	Increasing	LTMLE	16.08	13.47	1.193	0.969	1.469	0.096
		G-computation	17.17	12.67	1.356	1.103	1.667	0.004
	High stable	LTMLE	22.43	13.47	1.664	1.413	1.961	<0.001
		G-computation	21.01	12.67	1.658	1.386	1.982	<0.001
Model 2	Decreasing	LTMLE	14.50	13.52	1.072	0.865	1.329	0.524
		G-computation	15.87	13.55	1.171	0.956	1.435	0.128
	Increasing	LTMLE	16.29	13.52	1.205	0.981	1.479	0.075
		G-computation	17.31	13.55	1.277	1.047	1.558	0.016
	High stable	LTMLE	22.59	13.52	1.671	1.420	1.966	<0.001
		G-computation	20.75	13.55	1.531	1.294	1.813	<0.001
Model 3	Decreasing	LTMLE	14.58	13.90	1.049	0.851	1.293	0.655
		G-computation	15.36	13.04	1.178	0.954	1.456	0.129
	Increasing	LTMLE	15.95	13.90	1.147	0.938	1.403	0.180
		G-computation	16.24	13.04	1.246	1.015	1.529	0.036
	High stable	LTMLE	22.28	13.90	1.602	1.362	1.885	<0.001
		G-computation	19.30	13.04	1.481	1.232	1.780	<0.001
Model 4	Decreasing	LTMLE	14.52	14.92	0.973	0.812	1.167	0.770
		G-computation	14.97	13.85	1.081	0.899	1.299	0.409
	Increasing	LTMLE	16.70	14.92	1.120	0.946	1.326	0.189
		G-computation	17.23	13.85	1.243	1.047	1.476	0.013
	High stable	LTMLE	20.54	14.92	1.377	1.193	1.589	<0.001
		G-computation	18.84	13.85	1.360	1.160	1.594	<0.001

Model 5	Decreasing	LTMLE	14.70	14.66	1.003	0.821	1.226	0.976
		G-computation	16.08	14.19	1.133	0.934	1.375	0.204
	Increasing	LTMLE	15.96	14.66	1.089	0.898	1.321	0.386
		G-computation	17.20	14.19	1.212	1.003	1.463	0.046
	High stable	LTMLE	21.25	14.66	1.449	1.235	1.701	<0.001
		G-computation	19.62	14.19	1.383	1.167	1.638	<0.001

Model 1 adjust: age, gender; Model 2 adjust: age, gender, residence, education, marital, smoking, drinking; Model 3 adjust: age, gender, residence, education, marital, smoking, drinking, hypertension, dyslipidemia, cardiovascular disease, liver disease, chronic kidney disease; Model 4 adjust: age, gender, residence, education, marital, smoking, drinking, hypertension, dyslipidemia, cardiovascular disease, liver disease, chronic kidney disease, glucose, glycated hemoglobin;Model 5 adjust: age, gender, residence, education, marital, smoking, drinking, hypertension, dyslipidemia, cardiovascular disease, liver disease, chronic kidney disease, BMI; All covariates were assessed at baseline.

superlearner library 2 includes generalized linear Model, mean estimation, random forest, and xgboost.

Abbreviation: RR, Risk ratio; CI, Confidence interval; BMI, Body mass index.

Table S5. Landmark analysis of the associations between inflammatory status patterns and incident DM using ltmle.

		Standardised Marginal Risk For The Inflammatory Pattern(%)	Standardised Marginal Risk For The Low-stable Reference pattern(%)	RR	CI low	CI high	P value
Model 1	Decreasing	4.27	3.63	1.176	0.736	1.878	0.497
	Increasing	3.09	3.63	0.851	0.504	1.436	0.545
	High stable	4.99	3.63	1.374	0.933	2.026	0.108
Model 2	Decreasing	4.22	3.59	1.173	0.732	1.881	0.507
	Increasing	3.20	3.59	0.891	0.527	1.504	0.665

	High stable	5.16	3.59	1.436	0.972	2.121	0.069
Model 3	Decreasing	4.21	3.85	1.093	0.675	1.770	0.717
	Increasing	3.16	3.85	0.819	0.482	1.392	0.461
	High stable	5.17	3.85	1.341	0.894	2.011	0.156
Model 4	Decreasing	4.23	4.13	1.024	0.633	1.658	0.922
	Increasing	3.28	4.13	0.795	0.470	1.348	0.395
	High stable	4.72	4.13	1.143	0.760	1.720	0.520
Model 5	Decreasing	4.24	4.15	1.021	0.629	1.658	0.932
	Increasing	3.15	4.15	0.759	0.446	1.292	0.310
	High stable	5.02	4.15	1.209	0.799	1.828	0.369

Model 1 adjust: age, gender; Model 2 adjust: age, gender, residence, education, marital, smoking, drinking; Model 3 adjust: age, gender, residence, education, marital, smoking, drinking, hypertension, dyslipidemia, cardiovascular disease, liver disease, chronic kidney disease; Model 4 adjust: age, gender, residence, education, marital, smoking, drinking, hypertension, dyslipidemia, cardiovascular disease, liver disease, chronic kidney disease, glucose, glycated hemoglobin;Model 5 adjust: age, gender, residence, education, marital, smoking, drinking, hypertension, dyslipidemia, cardiovascular disease, liver disease, chronic kidney disease, BMI; All covariates were assessed at baseline.

Abbreviation: RR, Risk ratio; CI, Confidence interval; BMI, Body mass index.

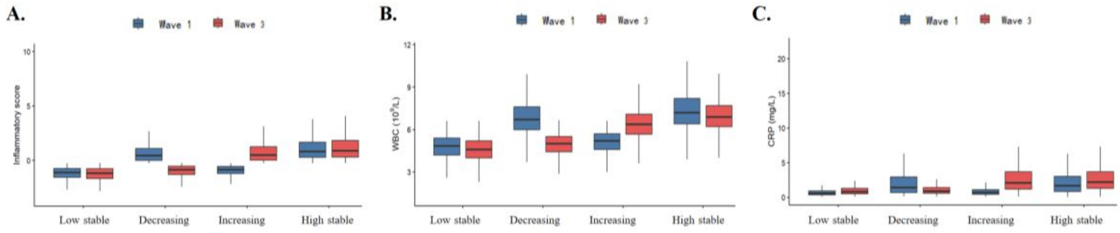

Supplementary Figure S1.Boxplots of inflammatory score, white blood cell count, and C-reactive protein levels across survey waves according to two-time-point inflammatory status patterns. WBC, white blood cell count; CRP, C-reactive protein.

Supplementary File S1：LTMLE model

The longitudinal data structure was specified as O=(W,A1,A2,Y). Here, W represented baseline covariates measured at Wave 1; A1and A2 represented inflammatory status measured at Waves 1 and 3, respectively, and were specified as the two treatment (exposure) nodes (Anodes); and Y represented incident diabetes and was specified as the outcome node (Ynodes). The joint values of A1 and A2 defined four exposure histories: low-stable (0,0), decreasing (1,0), increasing (0,1), and high-stable (1,1).

For each covariate adjustment model, the treatment mechanisms were specified as P(A1|W) and P(A2|A1,W), whereas the outcome mechanism was specified as E(Y|A1,A2,W). The same model-specific set of baseline covariates was included in both the treatment and outcome mechanisms, and these mechanisms were estimated using Super Learner. The different adjustment models therefore differed only in the baseline covariates included in W.No time-varying covariate nodes were specified (Lnodes = NULL) and no censoring nodes were specified (Cnodes = NULL).The target estimand was the contrast in standardized marginal risks of incident diabetes across four naturally occurring inflammatory exposure histories.

The Super Learner candidate library included a generalized linear model (SL.glm), a mean estimator (SL.mean), random forest (SL.randomForest), and XGBoost (SL.xgboost).Super Learner used 10-fold cross-validation to evaluate the cross-validated prediction risk of each candidate learner and assigned non-negative ensemble weights based on their cross-validation performance. The random forest learner comprised 1,000 trees.The minimum terminal node size was set to 1, with no restriction on the maximum number of terminal nodes. XGBoost was configured with 1,000 boosting iterations, a maximum tree depth of 4, a learning rate of 0.1, and a minimum child weight of 10. A binary logistic objective function and log-loss

evaluation metric were used, without early stopping.
